# The genome sequence of the Pied Smudge, *Ypsolopha sequella *(Clerck, 1759)

**DOI:** 10.12688/wellcomeopenres.18768.1

**Published:** 2023-01-19

**Authors:** Douglas Boyes, University of Oxford and Wytham Woods Genome Acquisition Lab, Darwin Tree of Life Barcoding collective, Wellcome Sanger Institute Tree of Life programme, Wellcome Sanger Institute Scientific Operations: DNA Pipelines collective, Tree of Life Core Informatics collective, William B.V. Langdon, Darwin Tree of Life Consortium

**Affiliations:** 1 UK Centre for Ecology and Hydrology, Wallingford, Oxfordshire, UK; 2 University of Oxford, Oxford, Oxfordshire, UK

**Keywords:** Ypsolopha sequella, Pied Smudge, genome sequence, chromosomal, Lepidoptera

## Abstract

We present a genome assembly from an individual *Ypsolopha sequella* (the Pied Smudge; Arthropoda; Insecta; Lepidoptera; Ypsolophidae). The genome sequence is 867 megabases in span. Most of the assembly is scaffolded into 30 chromosomal pseudomolecules with the Z sex chromosome assembled. The mitochondrial genome has also been assembled and is 15.3 kilobases in length. Gene annotation of this assembly on Ensembl identified 20,394 protein coding genes.

## Species taxonomy

Eukaryota; Metazoa; Ecdysozoa; Arthropoda; Hexapoda; Insecta; Pterygota; Neoptera; Endopterygota; Lepidoptera; Glossata; Ditrysia; Yponomeutoidea; Ypsolophidae; *Ypsolopha*; *Ypsolopha sequella* (Clerck, 1759) (NCBI:txid1870436).

## Background

*Ypsolopha sequella* (Clerck, 1759) is a micro moth of the family Yponomeutidae and the genus *Ypsolopha*. Adults in this group have relatively elongated forewings that are held close to the body at rest, which is often in a declining position. Of the 13 species in the genus known in Great Britain and Ireland ( Agassiz *et al.*, 2013), the adult *Y. sequella* is distinctive for the strongly contrasting black and white colouring in most specimens. Some have a suffusion of black scales in among the white ground colour, and can be quite dark, but the black markings along the dorsum remain visible in most specimens, including a rabbit-shaped blotch below the thorax, for which this moth is often affectionately known as the ‘bunny moth’ ( Wheeler, n.d.), although ( Porter, 2002) gives it the vernacular ‘Pied Smudge’.

In the UK, adults are on the wing between July and October ( Sterling & Parsons, 2018), with a peak in sightings in August ( Wheeler, no date). Adults are mostly nocturnal and are attracted to light, though rarely in numbers ( Sterling & Parsons, 2012). They can also occasionally be found around the larval foodplants by day, resting high up on the leaves ( Langmaid *et al.*, 2018), or dislodged lower down on trunks ( Agassiz, 1996). These foodplants are primarily Field maple ( *Acer campestre*) but also Sycamore ( *Acer psudoplantus*). While some *Ypsolopha* species overwinter as adults, *Y. sequella* hibernates as an egg on the twigs of these trees ( Agassiz, 1996). The greenish larva, which has the characteristic spindle shape of the genus, can then be found in a flimsy spinning on the leaves in May and June ( Agassiz, 1996; Langmaid *et al.*, 2018).

Both *A. campestre* and *A. pseudoplantus* are frequently grown ornamentally, and the moth can therefore be found where they occur in woodland and where they have been planted in suburban areas ( Agassiz, 1996). This planting may have aided the recent expansion of the moth, as it is thought to have for other species feeding on the same plants, such as Maple Prominent ( *Ptilodon cucullina*) ( Randle *et al.*, 2019; Waring *et al.*, 2017). Indeed, *Y. sequella* is one of several moth species expanding rapidly northwards into Scotland ( Emmet *et al.*, no date). Having first been recorded in the country in 1975 and then again in 1997 ( Bland, 1998), it is now resident in a few southern counties ( Knowler, 2012). Field Maple is scarcer in Scotland than in southern England, and here *Y. sequella* may depend on the more widespread Sycamore ( Sterling & Parsons, 2018). Away from Scotland, the moth is common in southern England ( Davis, 2012) and found throughout northern and central Europe into the Middle East ( Agassiz, 1996).

The genome of *Y. sequella* was sequenced as part of the Darwin Tree of Life Project, a collaborative effort to sequence all named eukaryotic species in the Atlantic Archipelago of Britain and Ireland. Here we present a chromosomally complete genome sequence for *Ypsolopha sequella*, based on two specimens from Wytham Woods, Oxfordshire, UK.

### Genome sequence report

**Figure 1.**
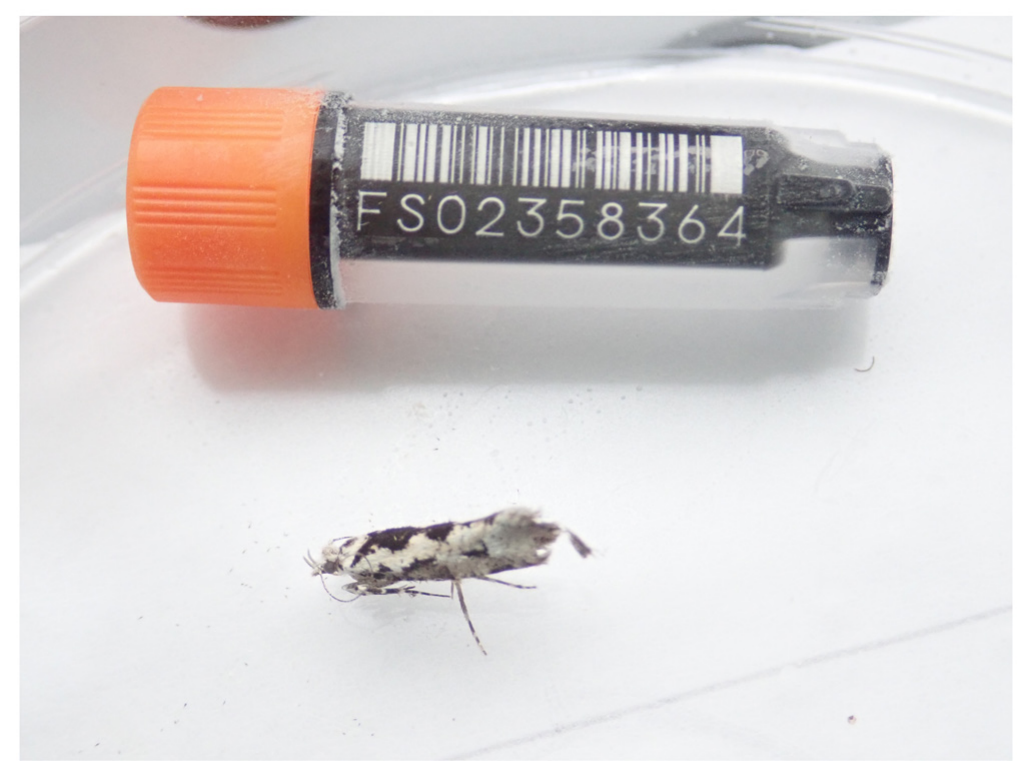
Image of the *Ypsolopha sequella* (ilYpsSequ2) specimen used for genome sequencing.

The genome was sequenced from one male *Y. sequella* ( Figure 1) collected from Wytham, Oxfordshire (biological vice-county: Berkshire), UK (latitude 51.77, longitude –1.33). A total of 27-fold coverage in Pacific Biosciences single-molecule HiFi long reads was generated. Primary assembly contigs were scaffolded with chromosome conformation Hi-C data. Manual assembly curation corrected 39 missing or mis-joins and removed seven haplotypic duplications, reducing the assembly length by 0.53% and the scaffold number by 6.83%.

**Figure 2.**
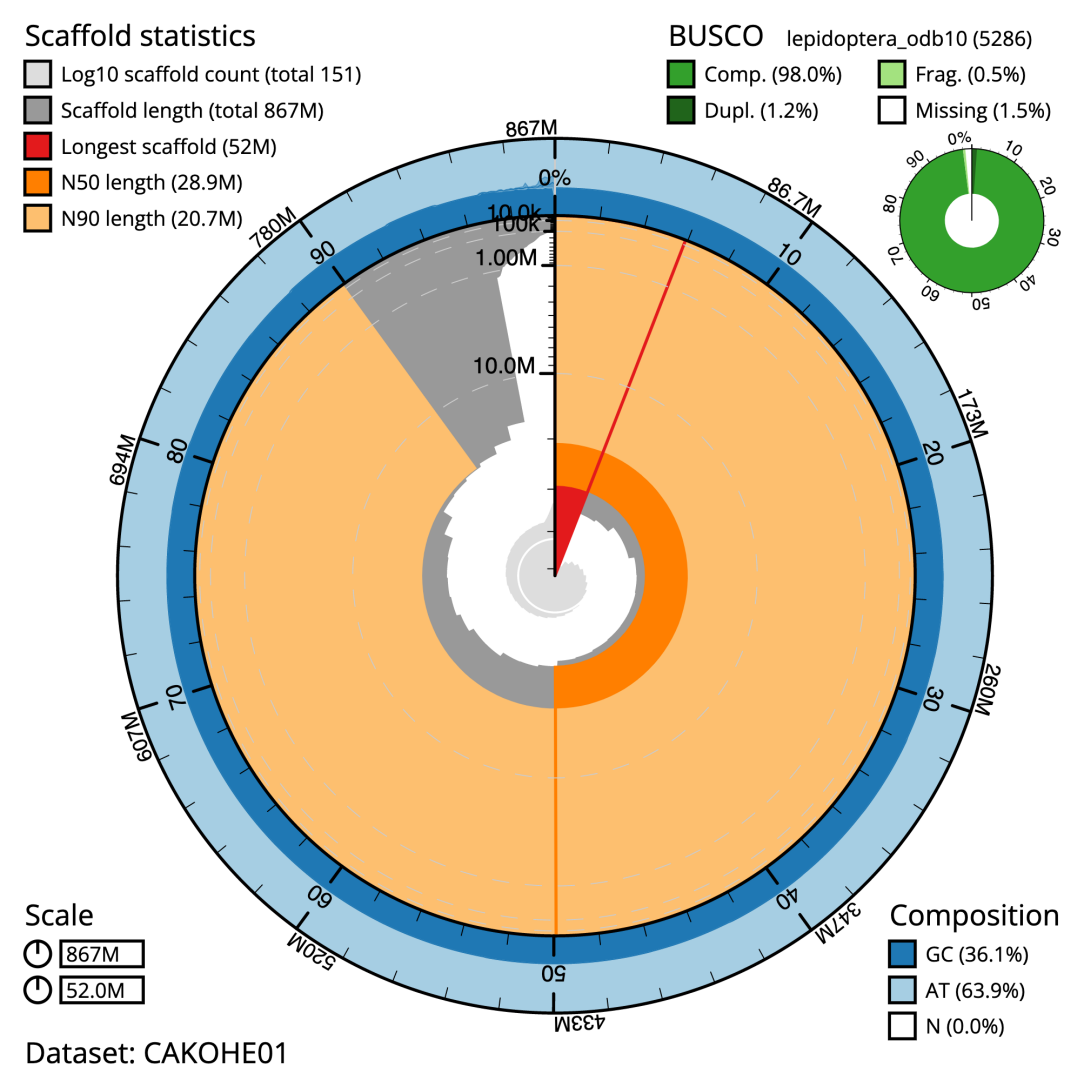
Genome assembly of *Ypsolopha sequella*, ilYpsSequ2.1: metrics. The BlobToolKit Snailplot shows N50 metrics and BUSCO gene completeness. The main plot is divided into 1,000 size-ordered bins around the circumference with each bin representing 0.1% of the 866,879,050 bp assembly. The distribution of sequence lengths is shown in dark grey with the plot radius scaled to the longest sequence present in the assembly (51,978,369 bp, shown in red). Orange and pale-orange arcs show the N50 and N90 sequence lengths (28,919,749 and 20,718,692 bp), respectively. The pale grey spiral shows the cumulative sequence count on a log scale with white scale lines showing successive orders of magnitude. The blue and pale-blue area around the outside of the plot shows the distribution of GC, AT and N percentages in the same bins as the inner plot. A summary of complete, fragmented, duplicated and missing BUSCO genes in the lepidoptera_odb10 set is shown in the top right. An interactive version of this figure is available at https://blobtoolkit.genomehubs.org/view/ilYpsSequ2.1/dataset/CAKOHE01/snail.

The final assembly has a total length of 866.9 Mb in 150 sequence scaffolds with a scaffold N50 of 28.9 Mb ( Table 1). Most (96.87%) of the assembly sequence was assigned to 30 chromosomal-level scaffolds, representing 29 autosomes and the Z sex chromosome ( Figure 2– Figure 5; Table 2). Chromosome-scale scaffolds confirmed by the Hi-C data are named in order of size. The assembly has a BUSCO v5.3.2 ( Manni *et al.*, 2021) completeness of 98% (single 96.9%, duplicated 1.2%) using the OrthoDB v10 lepidoptera reference set. While not fully phased, the assembly deposited is of one haplotype. Contigs corresponding to the second haplotype have also been deposited.

**Table 1.** Genome data for *Ypsolopha sequella*, ilYpsSequ2.1.

Project accession data		
Assembly identifier	ilYpsSequ2.1	
Species	*Ypsolopha sequella*	
Specimen	ilYpsSequ2	
NCBI taxonomy ID	1870436	
BioProject	PRJEB50740	
BioSample ID	SAMEA7519929	
Isolate information	ilYpsSequ2 (PacBio), ilYpsSequ1 (Hi-C, 10X)	
Assembly metrics *		*Benchmark*
Consensus quality (QV)	65.2	*≥ 50*
*k*-mer completeness	100	*≥ 95%*
BUSCO **	C:98.0%[S:96.9%,D:1.2%], F:0.5%,M:1.5%,n:5,286	*C ≥ 95%*
Percentage of assembly mapped to chromosomes	96.87%	*≥ 95%*
Sex chromosomes	Z	*localised homologous pairs*
Organelles	Mitochondrial genome assembled	*complete single alleles*
Raw data accessions		
PacificBiosciences SEQUEL II	ERR9081700, ERR9081701	
10X Genomics Illumina	ERR8571658–ERR8571661	
Hi-C Illumina	ERR8571662	
Genome assembly		
Assembly accession	GCA_934047225.1	
*Accession of alternate haplotype*	GCA_934041175.1	
Span (Mb)	866.9	
Number of contigs	228	
Contig N50 length (Mb)	20.8	
Number of scaffolds	150	
Scaffold N50 length (Mb)	28.9	
Longest scaffold (Mb)	52.0	
Genome annotation		
Number of protein-coding genes	20,394	

* Assembly metric benchmarks are adapted from column VGP-2020 of “ Table 1: Proposed standards and metrics for defining genome assembly quality” from ( Rhie *et al.* 2021).

** BUSCO scores based on the lepidoptera_odb10 BUSCO set using v5.3.2. C = complete [S = single copy, D = duplicated], F = fragmented, M = missing, n = number of orthologues in comparison. A full set of BUSCO scores is available at https://blobtoolkit.genomehubs.org/view/ilYpsSequ2.1/dataset/CAKOHE01/busco.

**Figure 3.**
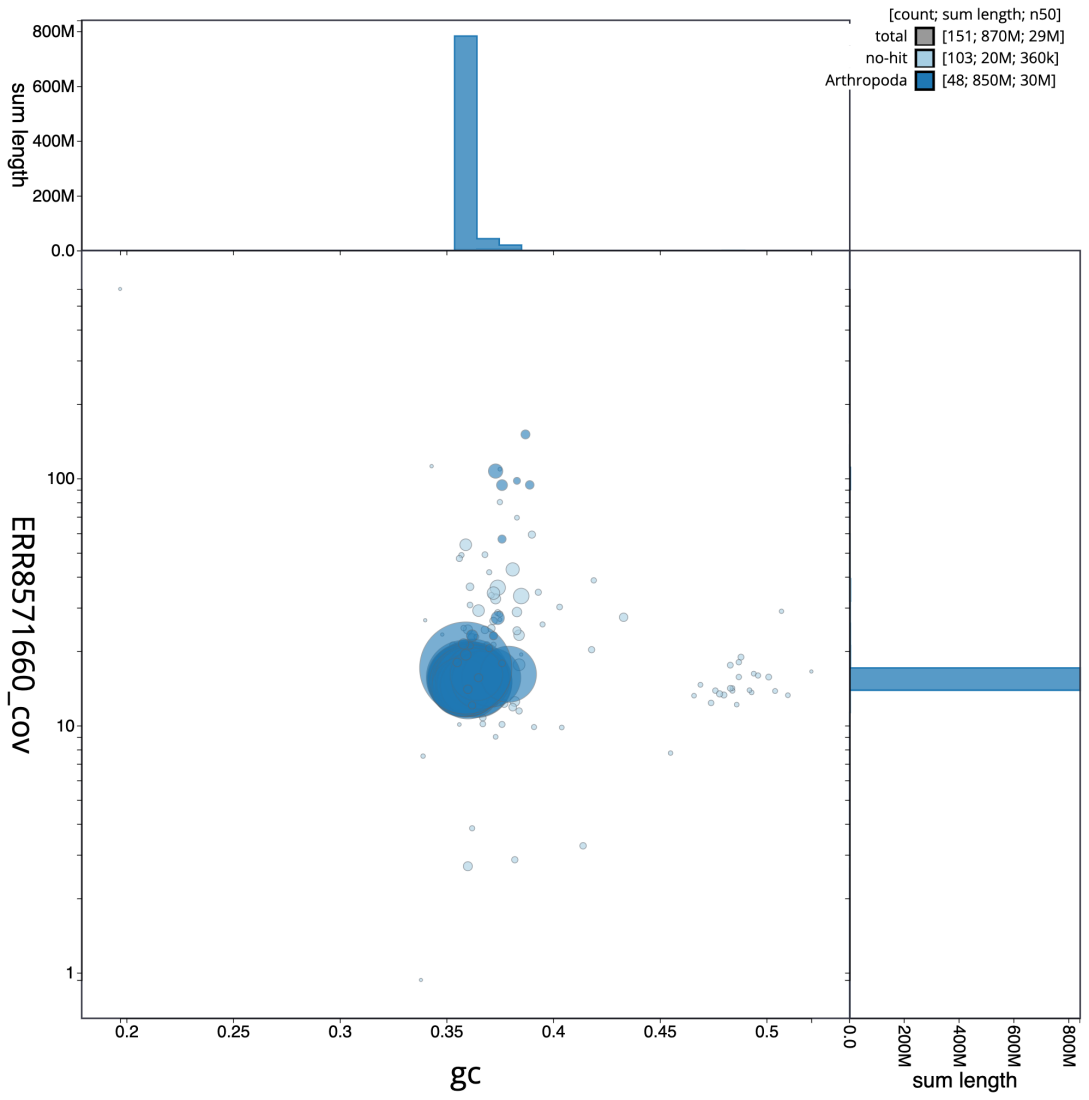
Genome assembly of *Ypsolopha sequella*, ilYpsSequ2.1: GC coverage. BlobToolKit GC-coverage plot. Scaffolds are coloured by phylum. Circles are sized in proportion to scaffold length. Histograms show the distribution of scaffold length sum along each axis. An interactive version of this figure is available at https://blobtoolkit.genomehubs.org/view/ilYpsSequ2.1/dataset/CAKOHE01/blob.

**Figure 4.**
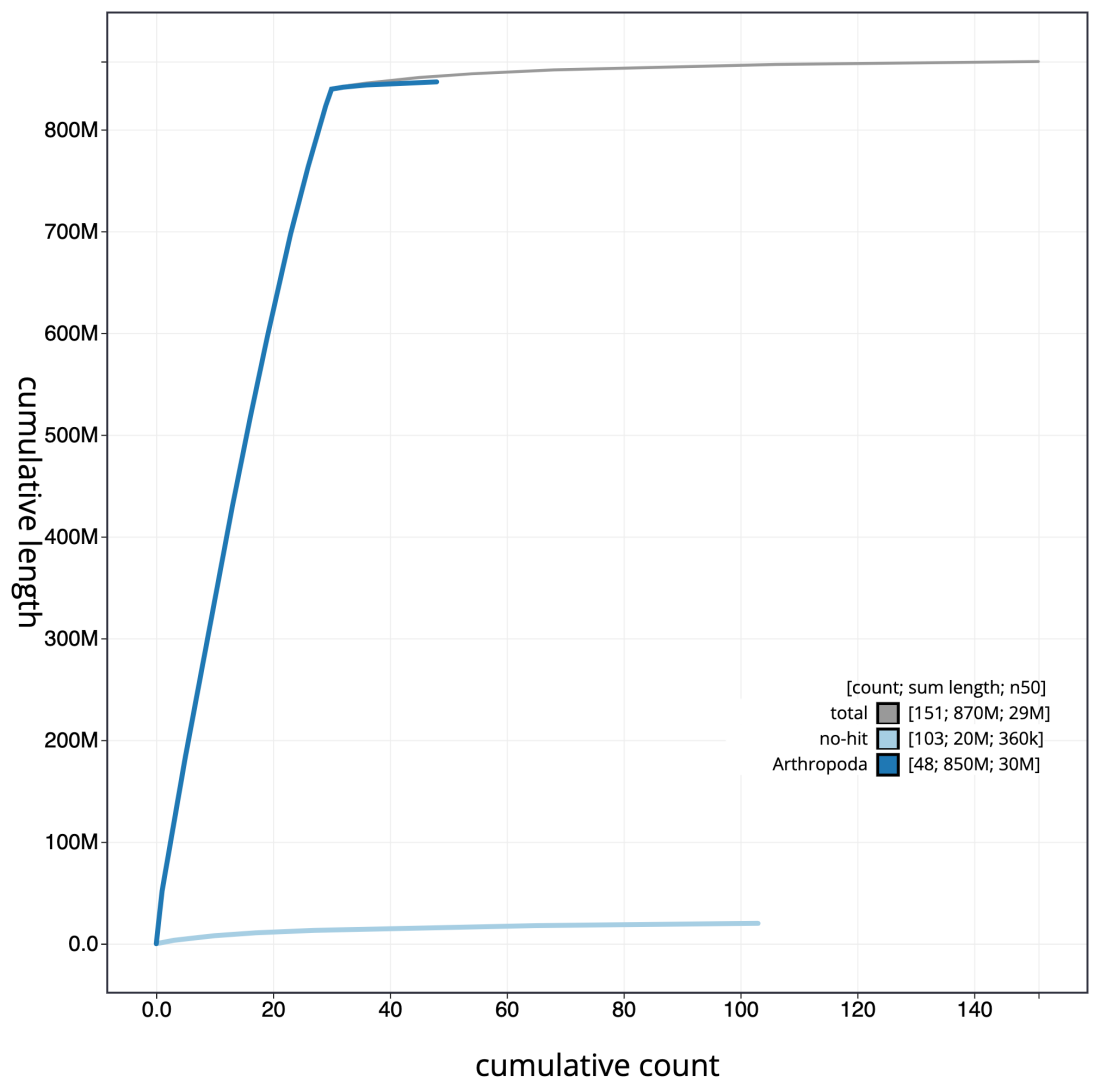
Genome assembly of *Ypsolopha sequella*, ilYpsSequ2.1: cumulative sequence. BlobToolKit cumulative sequence plot. The grey line shows cumulative length for all scaffolds. Coloured lines show cumulative lengths of scaffolds assigned to each phylum using the buscogenes taxrule. An interactive version of this figure is available at https://blobtoolkit.genomehubs.org/view/ilYpsSequ2.1/dataset/CAKOHE01/cumulative.

**Figure 5.**
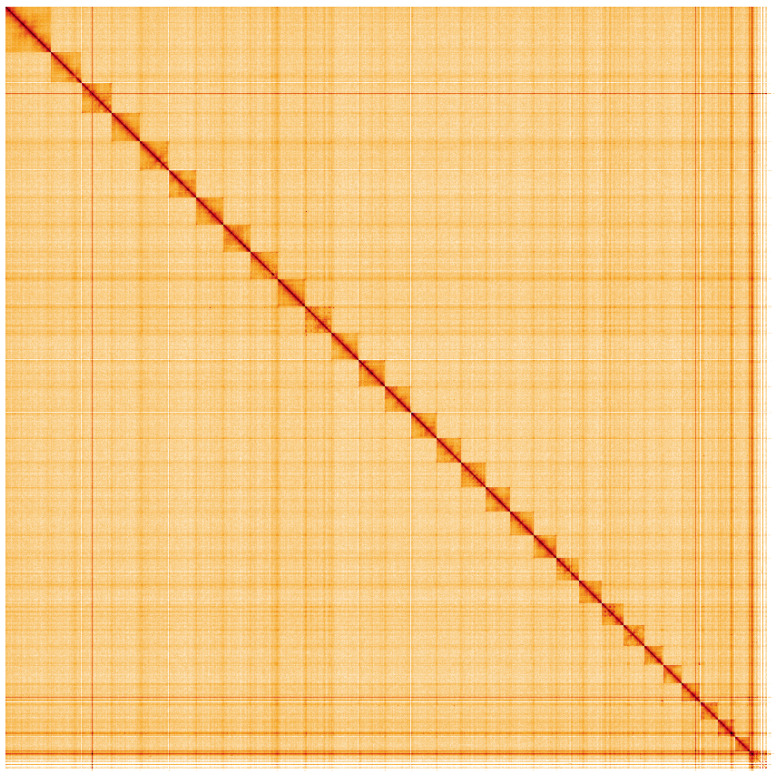
Genome assembly of *Ypsolopha sequella*, ilYpsSequ2.1: Hi-C contact map. Hi-C contact map of the ilYpsSequ2.1 assembly, visualised using HiGlass. Chromosomes are shown in order of size from left to right and top to bottom. An interactive version of this figure may be viewed at https://genome-note-higlass.tol.sanger.ac.uk/l/?d=A3UWotGzQ8WyAcqeFNsymw.

**Table 2.** Chromosomal pseudomolecules in the genome assembly of *Ypsolopha sequella*, ilYpsSequ2.

INSDC accession	Chromosome	Size (Mb)	GC%
OW203984.1	1	34.25	35.9
OW203985.1	2	33.72	35.8
OW203986.1	3	32.19	35.9
OW203987.1	4	32.19	36.2
OW203988.1	5	30.93	35.8
OW203989.1	6	30.85	36.1
OW203990.1	7	30.71	35.8
OW203991.1	8	30.67	36.4
OW203992.1	9	30.63	36.2
OW203993.1	10	30.5	36.4
OW203994.1	11	30.26	35.9
OW203995.1	12	30.09	36.1
OW203996.1	13	28.92	36.1
OW203997.1	14	28.51	35.7
OW203998.1	15	28.44	35.8
OW203999.1	16	27.53	35.8
OW204000.1	17	27.51	35.9
OW204001.1	18	26.48	35.9
OW204002.1	19	25.68	36
OW204003.1	20	25.43	36
OW204004.1	21	25.27	36
OW204005.1	22	25.1	36.4
OW204006.1	23	23.3	36
OW204007.1	24	21.35	36.1
OW204008.1	25	21.15	35.7
OW204009.1	26	20.72	37.1
OW204010.1	27	20.15	36.6
OW204011.1	28	18.82	37.9
OW204012.1	29	16.43	36.4
OW203983.1	Z	51.98	35.9
OW204013.1	MT	0.02	19.9

### Genome annotation report

The *Y. sequella* genome assembly (GCA_934047225.1) was annotated using the Ensembl rapid annotation pipeline ( Table 1; https://rapid.ensembl.org/Ypsolopha_sequella_GCA_934047225.1/). The resulting annotation includes 20,557 transcribed mRNAs from 20,394 protein-coding genes.

## Methods

### Sample acquisition and nucleic acid extraction

Two *Y. sequella* specimens (ilYpsSequ1 and ilYpsSequ2) were collected from the main track in Wytham, Oxfordshire (biological vice-county: Berkshire), UK (latitude 51.77, longitude –1.33) by Douglas Boyes, using a light trap. The specimens were identified by Douglas Boyes and snap-frozen on dry ice.

DNA was extracted at the Tree of Life laboratory, Wellcome Sanger Institute (WSI). The ilYpsSequ1 and ilYpsSequ2 specimens were weighed and dissected on dry ice, with tissue from ilYpsSequ1 set aside for Hi-C sequencing. Whole body tissue was disrupted using a Nippi Powermasher fitted with a BioMasher pestle. High molecular weight (HMW) DNA was extracted using the Qiagen MagAttract HMW DNA extraction kit. Low molecular weight DNA was removed from a 20 ng aliquot of extracted DNA using 0.8X AMpure XP purification kit prior to 10X Chromium sequencing; a minimum of 50 ng DNA was submitted for 10X sequencing. HMW DNA was sheared into an average fragment size of 12–20 kb in a Megaruptor 3 system with speed setting 30. Sheared DNA was purified by solid-phase reversible immobilisation using AMPure PB beads with a 1.8X ratio of beads to sample to remove the shorter fragments and concentrate the DNA sample. The concentration of the sheared and purified DNA was assessed using a Nanodrop spectrophotometer and Qubit Fluorometer and Qubit dsDNA High Sensitivity Assay kit. Fragment size distribution was evaluated by running the sample on the FemtoPulse system.

### Sequencing

Pacific Biosciences HiFi circular consensus (ilYpsSequ2) and 10X Genomics read cloud (ilYpsSequ1) DNA sequencing libraries were constructed according to the manufacturers’ instructions. DNA sequencing was performed by the Scientific Operations core at the WSI on Pacific Biosciences SEQUEL II (HiFi) and HiSeq X Ten (10X) instruments. Hi-C data were also generated from ilYpsSequ1 using the Arima v2 kit and sequenced on the HiSeq X Ten instrument.

### Genome assembly

Assembly was carried out with Hifiasm ( Cheng *et al.*, 2021) and haplotypic duplication was identified and removed with purge_dups ( Guan *et al.*, 2020). One round of polishing was performed by aligning 10X Genomics read data to the assembly with Long Ranger ALIGN, calling variants with freebayes ( Garrison & Marth, 2012). The assembly was then scaffolded with Hi-C data ( Rao *et al.*, 2014) using YaHS ( Zhou *et al.*, 2022). The assembly was checked for contamination as described previously ( Howe *et al.*, 2021). Manual curation was performed using HiGlass ( Kerpedjiev *et al.*, 2018) and Pretext ( Harry, 2022). The mitochondrial genome was assembled using MitoHiFi ( Uliano-Silva *et al.*, 2021), which performed annotation using MitoFinder ( Allio *et al.*, 2020). The genome was analysed and BUSCO scores generated within the BlobToolKit environment ( Challis *et al.*, 2020). Table 3 contains a list of all software tool versions used, where appropriate.

**Table 3.** Software tools and versions used.

Software tool	Version	Source
BlobToolKit	3.4.0	Challis *et al.*, 2020
freebayes	1.3.1-17- gaa2ace8	Garrison & Marth, 2012
Hifiasm	0.16.1-r375	Cheng *et al.*, 2021
HiGlass	1.11.6	Kerpedjiev *et al.*, 2018
Long Ranger ALIGN	2.2.2	https://support.10xgenomics. com/genome-exome/ software/pipelines/latest/ advanced/other-pipelines
MitoHiFi	2	Uliano-Silva *et al.*, 2021
PretextView	0.2	Harry, 2022
purge_dups	1.2.3	Guan *et al.*, 2020
YaHS	yahs-1.1.91eebc2	Zhou, McCarthy and Durbin, 2022

### Genome annotation

The BRAKER2 pipeline ( Brůna *et al.*, 2021) was used in the default protein mode to generate annotation for the *Ypsolopha sequella* assembly (GCA_934047225.1) in Ensembl Rapid Release.

### Ethics/compliance issues

The materials that have contributed to this genome note have been supplied by a Darwin Tree of Life Partner. The submission of materials by a Darwin Tree of Life Partner is subject to the Darwin Tree of Life Project Sampling Code of Practice. By agreeing with and signing up to the Sampling Code of Practice, the Darwin Tree of Life Partner agrees they will meet the legal and ethical requirements and standards set out within this document in respect of all samples acquired for, and supplied to, the Darwin Tree of Life Project. Each transfer of samples is further undertaken according to a Research Collaboration Agreement or Material Transfer Agreement entered into by the Darwin Tree of Life Partner, Genome Research Limited (operating as the Wellcome Sanger Institute), and in some circumstances other Darwin Tree of Life collaborators.

## Data Availability

European Nucleotide Archive: *Ypsolopha sequella* (pied smudge). Accession number PRJEB50740; https://identifiers.org/ena.embl/PRJEB50740 ( Wellcome Sanger Institute, 2022). The genome sequence is released openly for reuse. The *Ypsolopha sequella* genome sequencing initiative is part of the Darwin Tree of Life (DToL) project. All raw sequence data and the assembly have been deposited in INSDC databases. Raw data and assembly accession identifiers are reported in Table 1.

## Author information

Members of the University of Oxford and Wytham Woods Genome Acquisition Lab are listed here: https://doi.org/10.5281/zenodo.4789928.

Members of the Darwin Tree of Life Barcoding collective are listed here: https://doi.org/10.5281/zenodo.4893703.

Members of the Wellcome Sanger Institute Tree of Life programme are listed here: https://doi.org/10.5281/zenodo.4783585.

Members of Wellcome Sanger Institute Scientific Operations: DNA Pipelines collective are listed here: https://doi.org/10.5281/zenodo.4790455.

Members of the Tree of Life Core Informatics collective are listed here: https://doi.org/10.5281/zenodo.5013541.

Members of the Darwin Tree of Life Consortium are listed here: https://doi.org/10.5281/zenodo.4783558.
