# Supplementary material for: The genome sequence of the Pied Smudge, Ypsolopha sequella (Clerck, 1759)
Source: Wellcome Open Res. 2023 Jan 19;8:32. [Version 1] doi: 10.12688/wellcomeopenres.18768.1 (PMC10562790; doi:10.12688/wellcomeopenres.18768.1)
Supplement: Peer review history

# Reviewer report: Reviewer response for version 1

**DOI:** 10.21956/wellcomeopenres.20809.r61010

**Published:** 2024-07-15

**Authors:** Michal Rindoš

**Affiliations:** 1 Czech University of Life Sciences Prague, Prague, Czech Republic

The species description contains the basic information on the distribution and biology of the sequenced species. The description of the methods used to create this genome is practically the same as in the other species descriptions. The quality of the genome will most likely be excellent, as has been the case with other genomes.

Are sufficient details of methods and materials provided to allow replication by others?

Yes

Is the rationale for creating the dataset(s) clearly described?

Yes

Are the datasets clearly presented in a useable and accessible format?

Yes

Are the protocols appropriate and is the work technically sound?

Yes

Reviewer Expertise:

Biodiversity and systematics of Lepidoptera

I confirm that I have read this submission and believe that I have an appropriate level of expertise to confirm that it is of an acceptable scientific standard.

# Reviewer report: Reviewer response for version 1

**DOI:** 10.21956/wellcomeopenres.20809.r65130

**Published:** 2023-10-09

**Authors:** Guang Yang

**Affiliations:** 1 Fujian Agriculture and Forestry University, Fuzhou, Fujian, China

In the present article, the author provided the genome assembly of *Ypsolopha sequella* (the Pied Smudge; Arthropoda, Insecta, Lepidoptera, Ypsolophidae). The total genome size is 867 megabases with 30 chromosomes. Overall, the article is well-written and provides valuable information that could be further used. I just have a few minor suggestions.

1. It would be better to provide some more interpretation for the genome annotation either in terms of table(s) or figure(s).
2. It would be great if the author could provide the evolutionary relationship of *Ypsolopha sequella* with other lepidopteran species.
3. In the Background, please provide details on why the *Ypsolopha sequella* genome sequencing is necessary.
4. Please be consistent in using species names in the text, better to use abbreviated names such as *Y. sequella* .
5. Figures are blurry, better to use high-resolution pictures.
6. In the title of Table 2, the “ilYpsSequ2”  should be “ilYpsSequ2.1”.

Are sufficient details of methods and materials provided to allow replication by others?

Yes

Is the rationale for creating the dataset(s) clearly described?

Yes

Are the datasets clearly presented in a useable and accessible format?

Yes

Are the protocols appropriate and is the work technically sound?

Yes

Reviewer Expertise:

insect molecular biology

I confirm that I have read this submission and believe that I have an appropriate level of expertise to confirm that it is of an acceptable scientific standard.

# Reviewer report: Reviewer response for version 1

**DOI:** 10.21956/wellcomeopenres.20809.r57965

**Published:** 2023-06-02

**Authors:** Kay Lucek

**Affiliations:** 1 University of Basel, Basel, Switzerland

The authors present the chromosome level genome assembly of the micro moth *Ypsolopha sequella*. The assembly consists of 30 chromosomes including the Z chromosome, suggesting that a male individual was sequenced. Interestingly, a different individual was used to generate the Hi-C and 10X Chromium data. My only question about the presented note concerns this second individual – was it also a male and did the use of a different individual interfere with the assembly?

 Besides the W chromosome, the assembly is highly complete as suggested by the high BUSCO score. Overall, the presented assembly will be of great value to study genome as well as genome size evolution in Lepidoptera.

Are sufficient details of methods and materials provided to allow replication by others?

Yes

Is the rationale for creating the dataset(s) clearly described?

Yes

Are the datasets clearly presented in a useable and accessible format?

Yes

Are the protocols appropriate and is the work technically sound?

Yes

Reviewer Expertise:

Speciation genomics

I confirm that I have read this submission and believe that I have an appropriate level of expertise to confirm that it is of an acceptable scientific standard.
